# THE MINI-MENTAL STATE EXAMINATION IN A CHINESE POPULATION: RELIABILITY, VALIDITY, AND MEASUREMENT INVARIANCE

**DOI:** 10.1093/geroni/igad104.1275

**Published:** 2023-12-21

**Authors:** Bjoern Andersson, Hao Luo

**Affiliations:** University of Oslo, Oslo, Oslo, Norway; The University of Hong Kong, Hong Kong, Hong Kong

## Abstract

The Mini-Mental State Examination (MMSE) is a widely used screening tool for cognitive impairment and dementia in many cultures and regions. However, the properties of the MMSE in the Chinese context have not been evaluated with large population-representative samples. It is also unclear whether item properties of the MMSE differ across age and education subgroups. We study the psychometric properties of the MMSE using data from 8974 respondents aged 60 years or older in the 2018 Chinese Health and Retirement Longitudinal Study (CHARLS). Using item response theory (IRT) for the ordinal data, we confirm a three-dimensional factor structure for the MMSE in CHARLS in accordance with the underlying theoretical framework and establish acceptable model-data fit (SRMSR = 0.036). Total scores and subscale scores of the MMSE correlated positively with each other and correlated negatively with ADL (r = -0.19), IADL (-0.30), and CESD scores (-0.25). The reliability of the total MMSE scores was estimated at 0.78, indicating moderate measurement precision in the study population. Multiple group IRT analysis found that item characteristics and item functioning of 5 items differed by age group and 14 items differed by educational level. This analysis provided evidence on violations to measurement invariance of MMSE items, pointing to a need to account for item-level differences in measurement properties primarily with respect to education level when using the MMSE in CHARLS and, more generally, in Chinese populations. In addition, revised cognitive measures are advised to be used in CHARLS to improve the precision of measurement.

